# Editorial: Physiological computing of social cognition, volume II

**DOI:** 10.3389/fnhum.2023.1152291

**Published:** 2023-02-14

**Authors:** Antonio Fernández-Caballero, Amir Hussain, José Miguel Latorre, Arturo Martínez-Rodrigo, Roberto Rodriguez-Jimenez, Patricia Fernández-Sotos

**Affiliations:** ^1^Departamento de Sistemas Informáticos, Escuela Técnica Superior de Ingenieros Industriales, Universidad de Castilla-La Mancha, Albacete, Spain; ^2^Emotion and Neurocognition Unit, Instituto de Investigación en Informática de Albacete, Universidad de Castilla-La Mancha, Albacete, Spain; ^3^CIBERSAM (Biomedical Research Networking Centre in Mental Health), Madrid, Spain; ^4^Centre of AI and Data Science, Edinburgh Napier University, Edinburgh, United Kingdom; ^5^Departamento de Psicología, Facultad de Medicina, Universidad de Castilla-La Mancha, Albacete, Spain; ^6^Departamento de Sistemas Informáticos, Escuela Politécnica de Cuenca, Universidad de Castilla-La Mancha, Cuenca, Spain; ^7^Ingeniería Electrónica, Biomédica y de Telecomunicación, Instituto de Tecnologías Audiovisuales de Castilla-La Mancha, Universidad de Castilla-La Mancha, Cuenca, Spain; ^8^Servicio de Psiquiatría y Salud Mental, Hospital 12 de Octubre, Madrid, Spain; ^9^Departamento de Medicina Legal, Psiquiatría y Patología, Universidad Complutense de Madrid, Madrid, Spain; ^10^Servicio de Salud Mental, Complejo Hospitalario Universitario de Albacete, Servicio de Salud de Castilla-La Mancha, Albacete, Spain

**Keywords:** socio-cognitive computing, affective computing, social interaction, virtual and augmented reality, social robots, physiological computing

## Introduction

Social cognition addresses how people process, store, and use information about other people and social situations. It centers on the way in which cognitive processes play a role in our social interactions (Kunda, 1999; Hoppler et al., 2022). A growing interest has emerged in the connections between social cognition and brain function (Brothers, 1990). Although ample research has demonstrated that skills such as theory of mind, social perception, attributional style, and emotion perception are strongly related to social outcomes (Pinkham et al., 2016, 2018), research has been hampered by problems in measuring those abilities.

Social interactions have been explored on physiological measures for about a century. The physiological underpinnings of social constructs, processes, and behaviors have been extensively investigated, first by social psychophysiology and later by social neuroscience. Nowadays, physiological computing has arisen as a technology that uses electrophysiological data recorded directly from human activity to engage with a computing device (Fairclough, 2009; Fernández-Caballero et al., 2019). Such technology becomes even more relevant as computing gains ubiquity in daily environments.

“*Physiological Computing of Social Cognition*” should be understood as the application of physiological informatics to the assessment and/or treatment of social cognitive abilities (Chanel and Mühl, 2015; Fernández-Sotos et al., 2019). It comprises a range of interdisciplinary theoretical frameworks, methodologies, and hardware/software tools to interpret/act upon how human physiology mediates social interactions. This Volume II of the Research Topic provides a resource for researchers and scholars who currently pursue an interest in social cognitive deficit assessment and/or social cognitive training (e.g., cognitive enhancement therapies, attributional style training, social cognition and interaction training, or affect recognition training, among others). Papers with a particular focus on multidisciplinary approaches and multi-modality were especially welcome in this issue. In the end, a total of seven papers were accepted in this Research Topic.

## The papers

The first paper “*Greater Social Competence Is Associated With Higher Interpersonal Neural Synchrony in Adolescents With Autism*” by Key et al. addresses the difficulty of engaging in reciprocal social interactions for individuals with ASD. The authors conduct a hyper scanning electroencephalography study examining neural synchrony as a mechanism that supports interpersonal social interaction. Interpersonal neural synchrony was significantly greater during social interaction compared to baseline. Lower levels of synchrony were associated with increased behavioral symptoms of social difficulties.

A second paper by Fondevila et al., “*Subliminal Priming Effects of Masked Social Hierarchies During a Categorization Task: An Event-Related Brain Potentials Study*,” explores the evidence that status detection increases attentional resources. The authors examine the masked priming effects of social status cues during a categorization task. Event-related brain potentials (ERPs) were time-locked to the presentation of two types of artworks. ERP results indicate early attentional effects with larger amplitudes for processing artworks after high and military ranks. P3a increased for all artworks primed by religious vs. military figures.

Another article, “*EEG brain signals to detect the sleep health of a driver: An automated framework system based on deep learning*,” by Ettahiri et al. works on mental fatigue, a complex disorganization that affects human performance in work and daily activities. All traditional methods using electroencephalography to detect fatigue achieve low accuracy when a binary classification task is applied. In their study, a convolutional neural network is trained to recognize brain signals recorded by a wearable encephalographic cap, achieving an accuracy comparable to state-of-the-art methods.

In another article, “*Impaired sustained attention in groups at high risk for antisocial personality disorder: A contingent negative variation and standardized low-resolution tomographic analysis study*,” by Guan et al., the characteristics of contingent negative variation (CNV) in groups at high risk for antisocial personality disorder are explored. The CNV amplitude characteristics in a conduct disorder (CD) + antisocial personality (AP) group and the AP group were more consistent and fluctuated around baseline, indicating that the absence of attentional maintenance resulted in impairments in attentional allocation and motor preparation in these groups.

The next paper, “*Transcranial Direct Current Stimulation Over the Right Temporal Parietal Junction Facilitates Spontaneous Micro-Expression Recognition*,” by Ge et al. remind us that the accurate recognition and interpretation of micro-expressions enhances interpersonal interaction and social communication. In this study, the authors investigated the effects of training on micro-expression recognition and concluded that improved recognition accuracy of spontaneous fear micro-expressions was positively correlated with personal distress in the anodal group.

The paper “*A Spherical Phase Space Partitioning Based Symbolic Time Series Analysis (SPSPSTSA) for Emotion Recognition Using EEG Signals*” by Tavakkoli and Motie Nasrabadi introduces a new approach in symbolic time series analysis for signal phase space partitioning and symbol sequence generation. The authors present a topic-independent protocol to solve the generalization problem. The DEAP dataset was used, and high accuracies were achieved in classifying emotions, demonstrating that the proposed subject-independent method is more accurate than many other methods in various studies.

The last paper, “*Evaluating attention deficit hyperactivity disorder symptoms in children and adolescents through tracked head movements in a virtual reality classroom: The effect of social cues with different sensory modalities*,” by Cho et al. notes that there is a relative paucity of studies that focus on the cues that may influence the attention of children with ADHD in classroom settings. Children and adolescents with ADHD showed a decrease in task-irrelevant movements in the direction of the intended orientation in the presence of social cues using virtual reality.

## Conclusion

The seven articles altogether have attracted more than 8,500 visits as of January 27, 2022. This affirms the popularity of physiological computation of social cognition as a subject for the readership. This Research Topic's guest editors believe in the rising significance of physiological computation in such diverse domains as computer science, engineering, biology, psychology, medicine, and neuroscience. In addition, the guest editors also feel that physiological computing has not achieved its potential yet. Furthermore, the development of physiological computing applications in the field of social cognition still remains in its infancy (Fernández-Sotos et al., 2019; García et al., 2019). In the near future, a growth of solutions exploiting the social cognition/physiological computing binomial is anticipated.

## Author contributions

All authors listed have made a substantial, direct, and intellectual contribution to the work and approved it for publication.
